# miRNA‐210‐3p regulates trophoblast proliferation and invasiveness through fibroblast growth factor 1 in selective intrauterine growth restriction

**DOI:** 10.1111/jcmm.14335

**Published:** 2019-04-16

**Authors:** Lin Li, Xuan Huang, Zhiming He, Yuanyan Xiong, Qun Fang

**Affiliations:** ^1^ Department of Obstetrics and Gynecology, Fetal Medicine Center The First Affiliated Hospital of Sun Yat‐sen University Guangzhou Guangdong China; ^2^ Key Laboratory of Gene Engineering of the Ministry of Education and State Key Laboratory of Biocontrol, School of Life Sciences Sun Yat‐sen University Guangzhou Guangdong China

**Keywords:** fibroblast growth factor 1, miRNA‐210‐3p, monochorionic twins, selective intrauterine growth restriction, trophoblast

## Abstract

Selective intrauterine growth restriction (sIUGR), which affects approximately 10%‐15% of monochorionic (MC) twin pregnancies, is highly associated with intrauterine foetal death and neurological impairment in both twins. Data suggest that unequal sharing of the single placenta is the main contributor to birth weight discordance. While MC twins and their placenta derive from a single zygote and harbour almost identical genetic material, the underlying mechanisms of phenotypic discrepancies in MC twins remain unclear. MicroRNAs are small non‐coding RNA molecules that regulate gene expression but do not change the DNA sequence. Our preliminary study showed that microRNA‐210‐3p (miR‐210‐3p) was significantly upregulated in the placental share of the smaller sIUGR twin. Here, we investigate the potential role of miR‐210‐3p in placental dysplasia, which generally results from dysfunction of trophoblast cells. Functional analysis revealed that miR‐210‐3p, induced by hypoxia‐inducible factor 1α (HIF1α) under hypoxic conditions, suppressed the proliferation and invasiveness of trophoblast cell lines. Further RNA sequencing analysis and luciferase reporter assays were performed, revealing that fibroblast growth factor 1 (FGF1) is an influential target gene of miR‐210‐3p. Moreover, correlations among miR‐210‐3p levels, HIF1α and FGF1 expression and the smaller placental share were validated in sIUGR specimens. These findings suggest that upregulation of miR‐210‐3p may contribute to impaired placentation of the smaller twin by decreasing FGF1 expression in sIUGR.

## 1 INTRODUCTION

Twin pregnancies carry a higher risk of maternal and foetal complications than singleton pregnancies. Approximately 30% of twin pregnancies are monochorionic (MC),^1^ in which the foetuses derive from one fertilized zygote and share a single placenta. Selective intrauterine growth restriction (sIUGR), affecting approximately 10%‐15% of MC twin pregnancies, is defined by an estimated foetal weight (EFW) of one foetus below the 10th percentile with or without a difference in EFW of greater than 25% between the foetuses 1, 2 and is significantly associated with intrauterine foetal death and neurological impairment of both twins.^2^, ^4^, ^5^ Accumulating evidence has revealed that unequal sharing of the single placenta is the main contributor to the birthweight discordance between MC twins.^6^, ^7^ We recently reported a higher rate of smaller placental volumes and less vessel branching in the smaller of the sIUGR twins.^8^ Inadequate placental share and decreased perfusion lead to limitations in oxygen and nutrient transport to the foetus, resulting in intrauterine growth restriction in sIUGR pregnancies. However, the molecular mechanism underlying the pathophysiological processes of sIUGR has not been fully elucidated.

Monochorionic twins are fundamentally monozygotic, harbouring almost identical genetic material and sharing a similar intrauterine environment. Recently, epigenetic mechanisms have been widely studied in embryonic and placental development.^9^ Epigenetics, which includes microRNAs (miRNAs), is the study of heritable changes in gene function that do not entail changes in the DNA sequence. MicroRNAs are short endogenous oligonucleotides (18‐22 bp) that post‐transcriptionally regulate gene expression in many biological processes by repressing mRNA translation or accelerating mRNA degradation.^10^, ^11^ More than 500 different miRNAs are expressed in the human placenta, and some are involved in various pregnancy‐related disorders.^12^, ^13^ In the first stage of pregnancy, trophoblast cells invade the maternal endometrium and spiral arteries to establish the maternal‐foetal interaction.^15^ This invasion process is essential for successful embryonic implantation, placentation and uteroplacental vasculature establishment.^16^ Accumulating evidence has shown that aberrant miRNA expression can impair trophoblast function. However, the majority of studies are performed with singletons, in which the regulatory role of miRNAs might be affected by differences in genetic backgrounds among individuals. Thus, MC twins are considered an optimal model for investigating epigenome‐mediated molecular mechanisms by using co‐twins as internal controls.

In our preliminary study, we compared the miRNAs in the placental share between the smaller and larger twins of sIUGR pregnancies using RNA sequencing (RNA‐seq) technology. Fifteen miRNAs were identified to be significantly upregulated in the placental share of the smaller twins relative to their expression in that of the larger twins. Among these miRNAs, microRNA‐210‐3p (miR‐210‐3p) was one of the most highly expressed in the smaller twins. MiR‐210 is an important hypoxia‐inducible factor (HIF) in many human diseases.^17^ Hypoxia‐inducible factor, a heterodimer consisting of an oxygen‐sensitive subunit α and a constitutively active subunit β, mediates cellular responses to hypoxia by regulating transcriptional activation of a multitude of genes.^18^, ^19^ Under hypoxic conditions, hypoxia‐inducible factor 1α (HIF1α) binds to the hypoxia‐responsive element (HRE) in the miR‐210 promoter and induces miR‐210 expression. Previously, our group found that the level of HIF1α mRNA was significantly higher in the placental shares of sIUGR twins, especially of the growth‐restricted foetuses, than in the placentas of non‐sIUGR twins.^20^ We propose that increased miR‐210‐3p expression is correlated with the relatively higher level of HIF1α in the sIUGR placenta, contributing to the growth discordance between monozygotic twins.

Fibroblast growth factor 1 (FGF1) is a member of the FGF family that exhibits various biological activities by controlling fundamental cellular functions, including proliferation, differentiation, adhesion and morphogenesis.^21^ Studies have reported that FGF1, a ubiquitously expressed factor in different cell types, is involved in angiogenesis, wound healing, myocardial morphogenesis and some tumours.^22^, ^23^ During embryogenesis, FGFs play major roles in implantation, gastrulation, mesoderm induction and neural, circulatory and skeletal system formation.^25^, ^26^ However, few studies have investigated the pathological role of FGF1 in the setting of placental insufficiency and foetal growth restriction. In this study, we examined miR‐210‐3p expression in the placental shares of MC twins and investigated the potential role of miR‐210‐3p in the pathogenesis of sIUGR. Our results provide novel evidence that FGF1 is a direct target of hypoxia‐induced miR‐210‐3p and is involved in the regulation of HTR‐8/SVneo cell invasion and proliferation.

## 2 MATERIALS AND METHODS

### 2.1 Tissue specimens

This study was approved and supervized by the ethics committee of the First Affiliated Hospital of Sun Yat‐Sen University (Guangzhou, China), and informed consent was obtained from all study participants. Placentas were obtained immediately after delivery by elective caesarean section from monochorionic diamniotic (MCDA) twin pregnancies (sIUGR group and control group, n = 18 per group) delivered in this hospital. After the maternal and foetal membranes were removed, placental tissues around the umbilical cord insertion of each twin were collected. Villous portions were harvested and washed in ice‐cold saline (0.9% NaCl) three times to remove blood. Samples for RNA extraction were transferred into sterile tubes and stored at –80°C.

All twin pairs included were diagnosed as MCDA by pre‐natal ultrasound according to the American College of Obstetricians and Gynecologists guidelines. Monochorionic diamniotic twin pregnancies complicated by an EFW of one foetus below the 10th percentile and a growth discordance greater than 25% were recruited to the sIUGR group. Growth discordance was calculated as follows: ([weight of the larger twin − weight of the smaller twin]/weight of the larger twin). Normal MCDA twin pregnancies without sIUGR or other complications were included in the control group. Pregnancies complicated by twin‐to‐twin transfusion syndrome (TTTS), intrauterine foetal death, congenital anomalies, chromosomal abnormalities and maternal complications (such as hypertension, diabetes mellitus, cardiovascular disease, autoimmune disease or malignancies) were excluded.

### 2.2 Cell culture

The human extravillous trophoblast cell line HTR‐8/SVneo was purchased from the American Type Culture Collection (ATCC, Manassas, VA). HTR‐8/SVneo cells were cultured in RPMI 1640 medium (Gibco Life Technologies, Carlsbad, CA), supplemented with 10% foetal bovine serum (FBS, Gibco Life Technologies) and 1% penicillin/streptomycin, in humidified air at 37°C with 5% CO_2_. Hypoxic culturing was performed by incubating HTR‐8/SVneo cells in a hypoxic incubator (Eppendorf, Hamburg, Germany) for 48 hours at oxygen concentrations of 10%, 5% and 2%.

### 2.3 Plasmids, small interfering RNAs, virus production and cell transfection

The open reading frames (ORFs) of FGF1 were cloned into the pSin‐EF2‐puro lentiviral vector. The 3′‐untranslated region (UTR) of FGF1 and its corresponding mutant were amplified and cloned downstream of the luciferase gene in a modified pGL3 control vector (Promega, Madison, WI). Small interfering RNAs (siRNAs) targeting FGF1 and HIF1α were obtained from RiboBio Co., Ltd. (Guangzhou, China). Transfection of plasmids or siRNA oligonucleotides was performed using Lipofectamine 3000 reagent (Invitrogen, Waltham, MA) according to the manufacturer's instructions. Lentiviral vectors expressing miR‐210‐3p, the miR‐210‐3p sponge or the corresponding control vectors were obtained from ObiO Technology (Shanghai, China). Stable cell lines were generated via lentiviral infection for 2 days and selected with appropriate antibiotics for 10 days.

### 2.4 RNA extraction, reverse transcription and quantitative real‐time PCR (qRT‐PCR)

Total RNA was extracted from placental tissues or cultured cells using TRIzol (Invitrogen) following the manufacturer's instructions. Complementary DNA (cDNA) was synthesized with gene‐specific primers from 1 µg of total RNA using PrimeScript RT Master Mix (Takara, Japan). MiR‐210‐3p and U6 transcripts were synthesized using specific stem‐loop primers (RiboBio Co., Ltd). The expression levels of miR‐210‐3p and FGF1 and HIF1 mRNA were quantified using SYBR Green Master Mix (Roche, Basel, Switzerland) on a CFX96 Real‐Time PCR System (Bio‐Rad, Hercules, CA). U6 and β‐actin were used as the internal controls to normalize the miRNA and mRNA expression levels, respectively, in each sample, and the relative gene expression levels were calculated with the 2^−ΔΔCt^ method. The sequences of the specific primers were as follows: FGF1 forward, 5′‐CTCCCGAAGGATTAAACGACG‐3′; FGF1 reverse, 5′‐GTCAGTGCTGCCTGAATGCT‐3′; HIF1α forward 5′‐‐3′; HIF1α ′TCGGCTAGTTAGGGTACACTTC‐3′, β‐actin forward, 5′‐CATGTACGTTGCTATCCAGGC‐3′; and β‐actin reverse, 5′‐CTCCTTAATGTCACGCACGAT‐3′.

### 2.5 Western blotting

Western blotting (WB) was performed according to the protocol of a standard method described previously.^28^ Briefly, proteins were extracted from placental tissues or HTR‐8/SVneo cells using 1× SDS lysis buffer. Equal amounts of protein were loaded onto SDS‐PAGE gels and transferred to polyvinylidene difluoride (PVDF) membranes. Membranes were incubated with primary monoclonal antibodies against FGF1 (1:500, ab9588; Abcam), HIF1α (1:250, ab1; Abcam) and α‐tubulin (1:2000, ab18251; Abcam) overnight at 4°C. Then, relative protein levels were quantified by scanning densitometry, and the relative gray value of the protein bands was calculated by using α‐tubulin as the internal control.

### 2.6 Cell viability assay

Cells were diluted in medium, and 1 × 10^3^ cells in 200 μL were seeded in 96‐well plates (six plates per group) and incubated (37°C, 5% CO_2_). For each 24‐hour time point, the cells were supplemented with 25 μL of sterile MTT (3‐[4,5‐dimethylthiazol‐2‐yl]‐2,5‐diphenyltetrazolium bromide) dye (Sigma, Saint Louis, MO) and incubated for an additional 4 hours at 37°C. Then, the culture medium containing MTT was removed before 150 μL of dimethyl sulfoxide (DMSO, Sigma) was added to each well. After shaking at room temperature for 10 minutes, the absorbance of the stained cells was measured at 562 nm. The experiment was performed in triplicate to obtain the average optical density (OD).

### 2.7 Cell invasion assay

Cell invasion was assessed using a Transwell chamber assay (Corning Inc, New York, NY) with Matrigel‐coated (BD Biosciences, Bedford, MA) membranes. First, cells were suspended in medium and seeded in the upper chambers of 24‐well Transwell plates (1:3 dilution in medium, 50 μL/well) with a Matrigel‐coated membrane. Then, RPMI 1640 medium (500 mL) containing 10% FBS was added to the lower chamber of the Transwell plates as a chemoattractant. After incubation for 24 hours (37°C, 5% CO_2_), the upper surface of the insert was gently wiped with a cotton swab to remove non‐invading cells. Cells invading into the lower chamber were fixed, stained, photographed and quantified by counting in five random 100× magnification fields.

### 2.8 Luciferase reporter assay

For luciferase reporter assays, 2 × 10^4^ cells were seeded in triplicate in 48‐well plates and allowed to adhere for 24 hours. The indicated plasmids (wild‐type or mutant) plus 1 ng of pRL‐TK Renilla plasmid were cotransfected into the corresponding lentivirus‐infected cells using Lipofectamine 3000 reagent (Life Technologies). Forty‐eight hours after transfection, a dual luciferase reporter assay (Promega, Madison, WI) was performed according to the manufacturer's instructions.

### 2.9 RNA‐seq and bioinformatic analysis

Samples of total RNA from the placental tissues of six pairs of sIUGR twins were extracted for miRNA sequencing. MiRNA sequencing was carried out commercially by Annoroad Gene Technology Corporation following standard protocols. Briefly, small RNAs (18‐30 nt) were separated and enriched using a 15% PAGE gel. Libraries were prepared using a TruSeq Small RNA Sample Preparation Kit and sequenced on an Illumina HiSeq 2500 (50SE). Clean reads were mapped to the human genome using miRDeep2, and heat map analysis was performed with the R programming language.

Samples of total RNA from HTR‐8/SVneo cells overexpressing miR‐210 or the corresponding vector control were extracted for mRNA sequencing. mRNA sequencing was carried out commercially by Vazyme Biotech Corporation following standard Illumina HiSeq protocols. Clean reads were mapped to the human genome using Hista2, and further analysis, including the generation of volcano plots and heat maps, was performed with the R programming language.

### 2.10 Statistical analysis

Statistical analyses were carried out using spss version 21 (IBM Corporation, Armonk, NY) and GraphPad Prism 6. Data are expressed as the means ± SDs of at least three independent experiments. The Mann‐Whitney test or Student's *t* test was used to compare two independent samples. One‐way ANOVA and least significant difference (LSD) multiple comparison tests were used to compare the differences among groups. Spearman's correlation coefficients were used to examine the correlation between miR‐210‐3p expression and HIF1α and FGF1 expression. A *p*‐value of <0.05 was considered to indicate statistical significance.

## 3 RESULTS

### 3.1 MiR‐210‐3p expression is elevated in the placental share of the smaller foetus in sIUGR pregnancies

**Figure 1:**
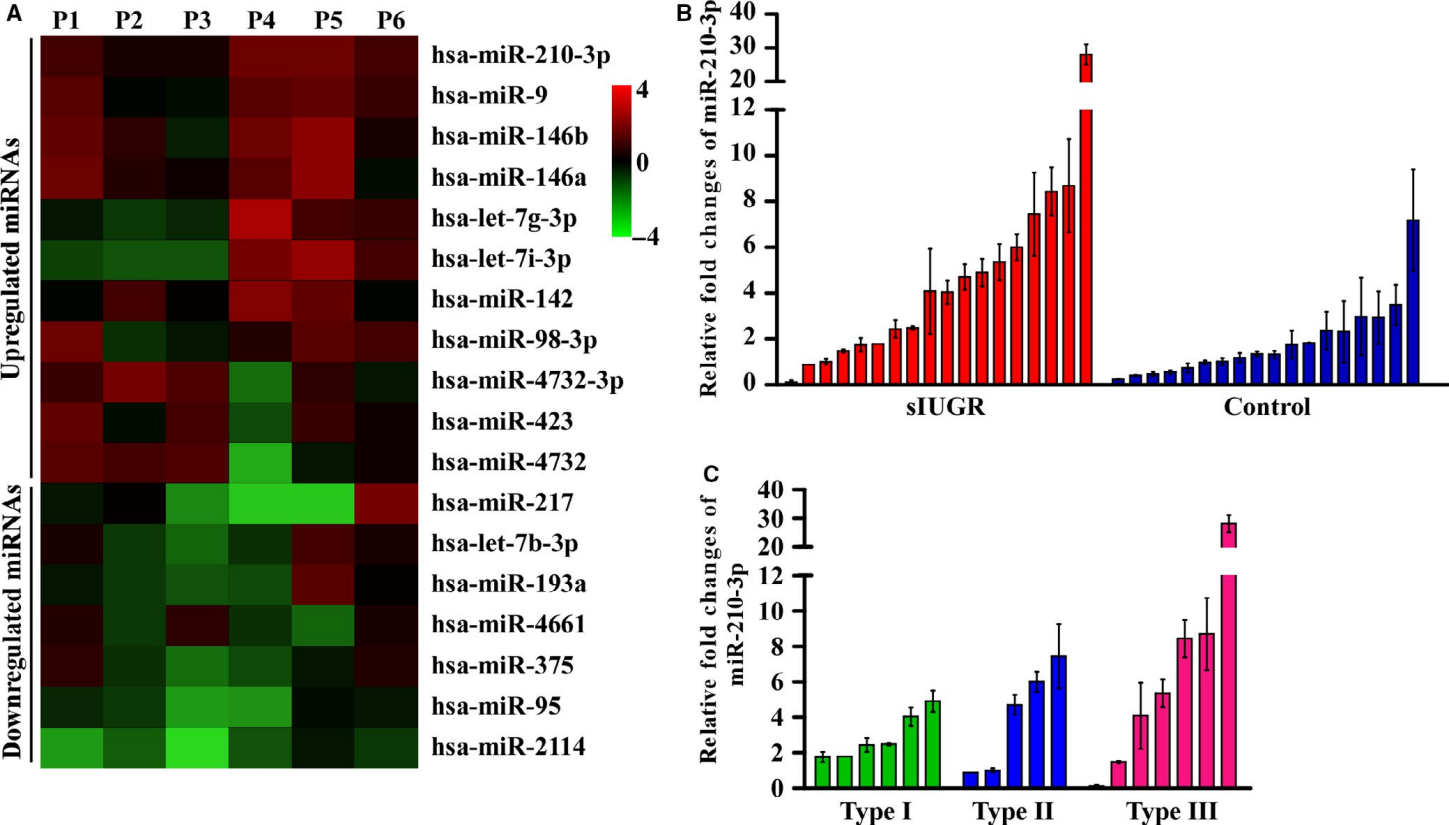
Expression of miR‐210‐3p in the placenta. A, Six paired (P1‐P6) selective intrauterine growth restriction (sIUGR) placental tissues were subjected to microRNA sequencing, and differentially expressed microRNAs between placental shares of the smaller twins and larger twins are shown on the heatmap. The scale colour bar represents log2 (fold change) between the smaller twins and larger twins. B, qRT‐PCR results showing miR‐210‐3p levels in the placental share of the smaller foetus compared to those of the larger foetus in the sIUGR group (n = 18) and control group (n = 18). C, miR‐210‐3p levels in the placental share of the smaller foetus compared to those of the larger foetus in three types of sIUGR twins

To screen for dysregulated miRNAs involved in sIUGR, we first compared differentially expressed miRNAs in placental shares of the larger twins with those in placental shares of the smaller twins through small RNA‐seq in six pairs of sIUGR twins. Eighteen differentially expressed miRNAs (11 upregulated miRNAs and seven downregulated miRNAs) were found (Figure 1A). Among these miRNAs, miR‐210‐3p was one of the most highly upregulated miRNAs (fold change = 3.6) in the placental share of smaller twins.

To verify the results of the microarray analysis, we assessed the relative expression of miR‐210‐3p in placental tissues obtained from the sIUGR group (n = 18) and control group (n = 18). The levels of miR‐210‐3p in the placental shares of each foetus were detected by qRT‐PCR. As shown in Figure 1B and Figure S1A, the average change in miR‐210‐3p expression in the placental shares of the smaller twins was 5.21‐fold [95% CI], 3.10‐ to 7.32‐fold], which was significantly higher than that in the placental shares of the larger twins in the sIUGR group (*P* < 0.01). In the control group, the average change in miR‐210‐3p expression in the placental shares of the smaller twins was 1.84‐fold (95% CI, 1.26‐ to 2.43‐fold) that of the larger twins.

The clinical characteristics of the two groups of MCDA twins are summarized in Table 1. No significant difference was found in maternal age between the sIUGR and control groups. The birth weights of both the smaller and larger foetuses were significantly lower in the sIUGR group than in the control group, and the birth weight discordance was significantly greater in the sIUGR group than in the control group (*P* < 0.01). Lower birth weight was closely associated with an earlier gestational age at delivery in the sIUGR group (average, 32 weeks) relative to that in the normal group (average, 35 weeks). The placental share of the smaller sIUGR twins (29.7%) was significantly less than that of the smaller twins in the control group (45.0%) (*P* < 0.01).

**Table 1:** Clinical characteristics of normal and sIUGR twin pregnancies

	Normal (n = 18)	sIUGR				*P* value^a^
		Total (n = 18)	Type I (n = 6)	Type II (n = 5)	Type III (n = 7)	
Maternal age at delivery (y)	30.4 ± 4.2	30.6 ± 6.0	31.3 ± 7.7	30.8 ± 6.1	29.9 ± 5.2	0.898
Gestational weeks at delivery (wk)	35.9 ± 1.0	32.4 ± 2.1	33.3 ± 0.7	32.0 ± 1.0	32.0 ± 0.9	<0.01
Birth weight of larger twins (kg)	2.5 ± 0.2	1.8 ± 0.4	1.9 ± 0.2	1.7 ± 0.2	1.8 ± 0.1	<0.01
Birth weight of smaller twins (kg)	2.4 ± 0.2	1.1 ± 0.3	1.2 ± 0.2	0.93 ± 0.1	1.2 ± 0.1	<0.01
Birth weight discordance (%)	5.3 ± 3.9	38.4 ± 11.6	39.6 ± 7.3	44.1 ± 3.9	33.2 ± 1.0	<0.01
Placenta share of smaller twins (%)	45.0 ± 7.0	29.7 ± 12.1	36.3 ± 5.1	23.4 ± 3.4	28.4 ± 4.9	<0.01

Data are shown as the mean ± SD.

Abbreviation: sIUGR, selective intrauterine growth restriction.

a Comparison between the normal group and sIUGR group.

Then, we examined whether the upregulation of miR‐210‐3p in the smaller twins was clinically relevant in sIUGR cases. A classification system based on the Doppler characteristics of the umbilical artery (UA) in the smaller sIUGR twin correlates well with distinct clinical evolution and outcomes.^29^ Monochorionic twins in the sIUGR group were classified into three types: type I, normal UA Doppler; type II, persistent absent or reversed end diastolic velocity flow (AREDF); or type III, intermittent AREDF (Table 1). The mean gestational age at delivery was earlier for type II and III twins than for type I twins. The placental share of the smaller type II (23.4%) and type III (28.4%) twins was smaller than that of the smaller type I twins (36.3%) (*P* < 0.01). Moreover, qRT‐PCR revealed that the mean fold change in miR‐210‐3p expression in type II and III twins was relatively higher than that in type I twins (Figure 1C).

### 3.2 Overexpression of miR‐210‐3p suppresses cell proliferation and invasiveness

**Figure 2:**
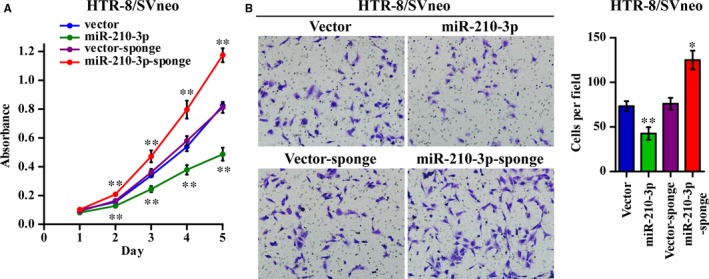
miR‐210‐3p inhibited HTR‐8/SVneo cell proliferation and invasiveness. HTR‐8/SVneo cells were stably transduced with miR‐210‐3p mimic, miR‐210‐3p sponge, or corresponding vector controls through lentivirus infection and antibiotic selection. A, Cell growth curves for the indicated cells were measured via an MTT assay. B, Representative images and quantification of invaded cells in the Matrigel‐coated Transwell assay are shown as indicated. (*P < 0.05, **P < 0.01)

Trophoblasts are the major structural and functional component of the human placenta. Given that miR‐210‐3p expression was increased in the placental shares of the smaller sIUGR foetuses, we next investigated the biological effect of miR‐210‐3p in trophoblasts. MicroRNA‐210‐3p, a miR‐210‐3p sponge, and corresponding control vectors were stably transfected into HTR‐8/SVneo cells via lentivirus. The transfection efficiency was confirmed via qRT‐PCR. Compared with negative control‐transfected cells, trophoblasts transfected with miR‐210‐3p lentivirus exhibited a significant decrease in growth, as analysed using MTT assays (Figure 2A). Moreover, Transwell assays showed that the invasive ability of miR‐210‐3p‐overexpressing HTR‐8/SVneo cells was drastically decreased. In contrast, inhibiting miR‐210‐3p markedly promoted the growth and invasive capabilities of HTR‐8/SVneo cells. Taken together, these data indicated that miR‐210‐3p suppressed cell proliferation and invasiveness, which consequently might lead to insufficient placentation.

### 3.3 Identification of FGF1 as a target of miR‐210‐3p in human trophoblast cells

**Figure 3:**
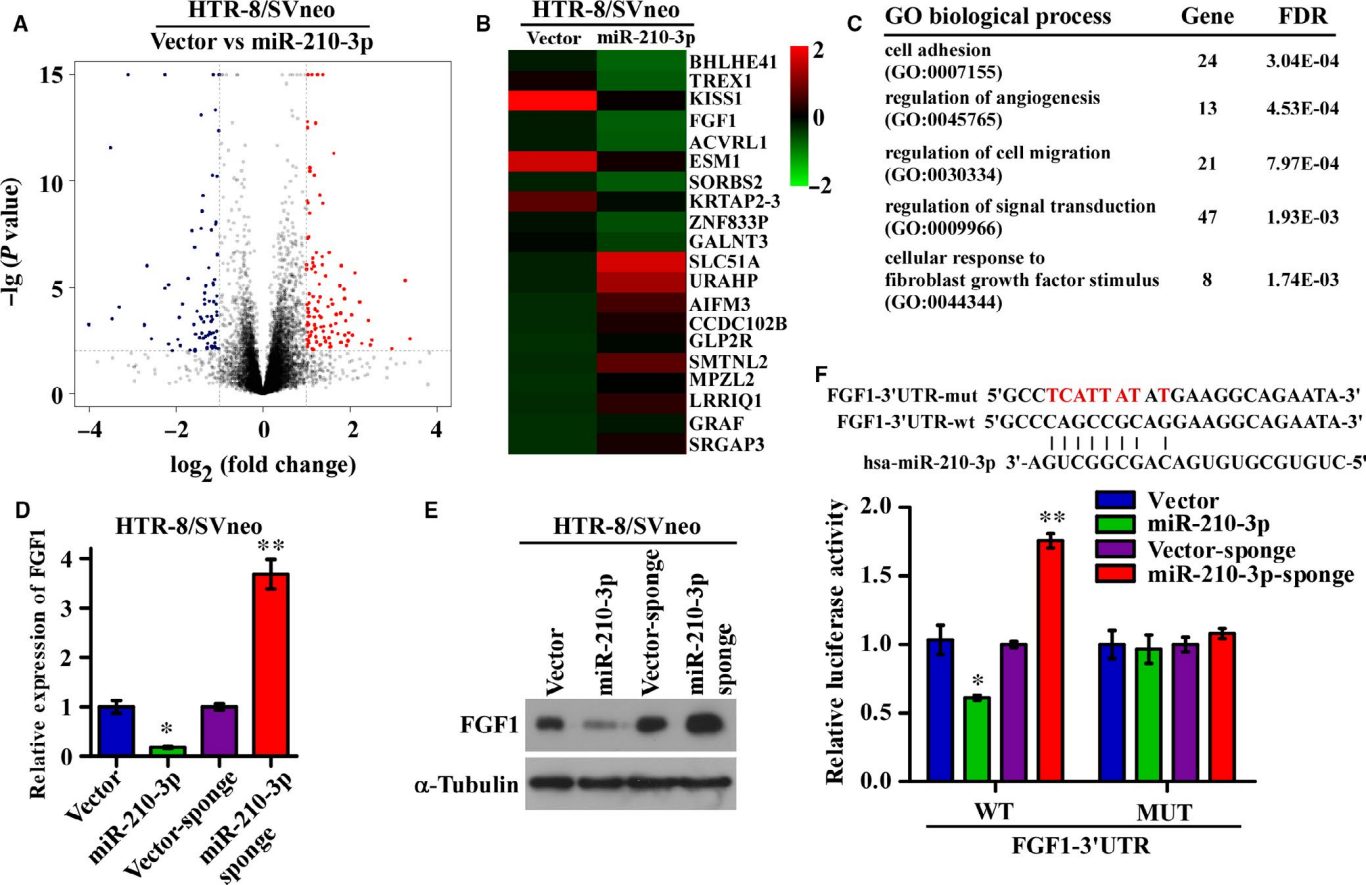
miR‐210‐3p directly targets fibroblast growth factor 1 (FGF1) in HTR‐8/SVneo cells. (A,B) HTR‐8/SVneo cells stably overexpressing miR‐210‐3p and corresponding vector control cells were subjected to mRNA sequencing. The differentially expressed genes were plotted, and the top 20 upregulated and downregulated genes are shown on the heatmap. The scale colour bar represents the normalized expression level in each sample. C, Differentially expressed genes were subjected to Gene Ontology (GO) biological process analysis, and the top five enriched gene sets are listed. D, qRT‐PCR analysis of the relative expression of FGF1 in the indicated cells. E, Western blotting analysis of the protein levels of FGF1 in the indicated cells. F, Schematic representation of the putative miR‐210‐3p binding site in the 3′‐untranslated region (3′‐UTR) of FGF1 and the corresponding mutant sequence are shown as indicated. These sequences were ligated into a luciferase reporter construct, and the results of the luciferase assay with reporters driven by the wild‐type and mutated FGF1 3′‐UTR in the indicated cells are shown. (*P < 0.05, **P < 0.01)

Because miRNAs regulate cellular processes by repressing target gene expression through interaction with the 3′‐UTR of mRNAs, we next explored the mechanism underlying the suppressive effect of miR‐210‐3p on the invasion and proliferation of trophoblasts. MicroRNA‐210‐3p‐overexpressing HTR‐8/SVneo cells and the corresponding control cells were subjected to RNA‐seq, and bioinformatic analysis showed that 139 genes were upregulated and 98 were downregulated in miR‐210‐3p‐overexpressing cells relative to their expression in the corresponding control cells (Figure 3A). The top 20 differentially expressed genes are listed, among which FGF1 was significantly downregulated in miR‐210‐3p‐overexpressing cells (Figure 3B). The differentially expressed genes were further clustered according to Gene Ontology (GO) biological processes, and the results showed that miR‐210‐3p mainly affected genes related to cell adhesion, angiogenesis, cell migration and fibroblast growth factor stimulation (Figure 3C). qRT‐PCR and WB analyses showed that FGF1 expression was significantly downregulated in miR‐210‐3p‐overexpressing cells and upregulated in cells transfected with the miR‐210‐3p inhibitor compared with that in the corresponding control cells (Figure 3D,E). By analyzing the FGF1 3′‐UTR sequence with the bioinformatics software miRWALK (http://mirwalk.umm.uni-heidelberg.de/), we found a potential binding site for miR‐210‐3p, indicating that FGF1 may be a direct target of miR‐210‐3p. Therefore, we constructed luciferase reporter plasmids containing FGF1 3′‐UTR sequences with the wild‐type or corresponding mutated miR‐210‐3p binding site. The reporter assays showed that compared to the relative luciferase activity in control cells, the relative luciferase activity from the wild‐type FGF1 3′‐UTR was significantly decreased in miR‐210‐3p‐overexpressing cells and increased in miR‐210‐3p‐inhibited (Figure 3F). However, when the predicted miR‐210‐3p binding sequence in the FGF1 3′‐UTR was mutated, the luciferase activity was no longer affected by miR‐210‐3p (Figure 3F). Collectively, these results validated that miR‐210‐3p directly suppressed FGF1 expression in HTR‐8/SVneo trophoblast cell lines.

### 3.4 miR‐210‐3p regulates trophoblast invasiveness and proliferation through a mechanism involving FGF1

**Figure 4:**
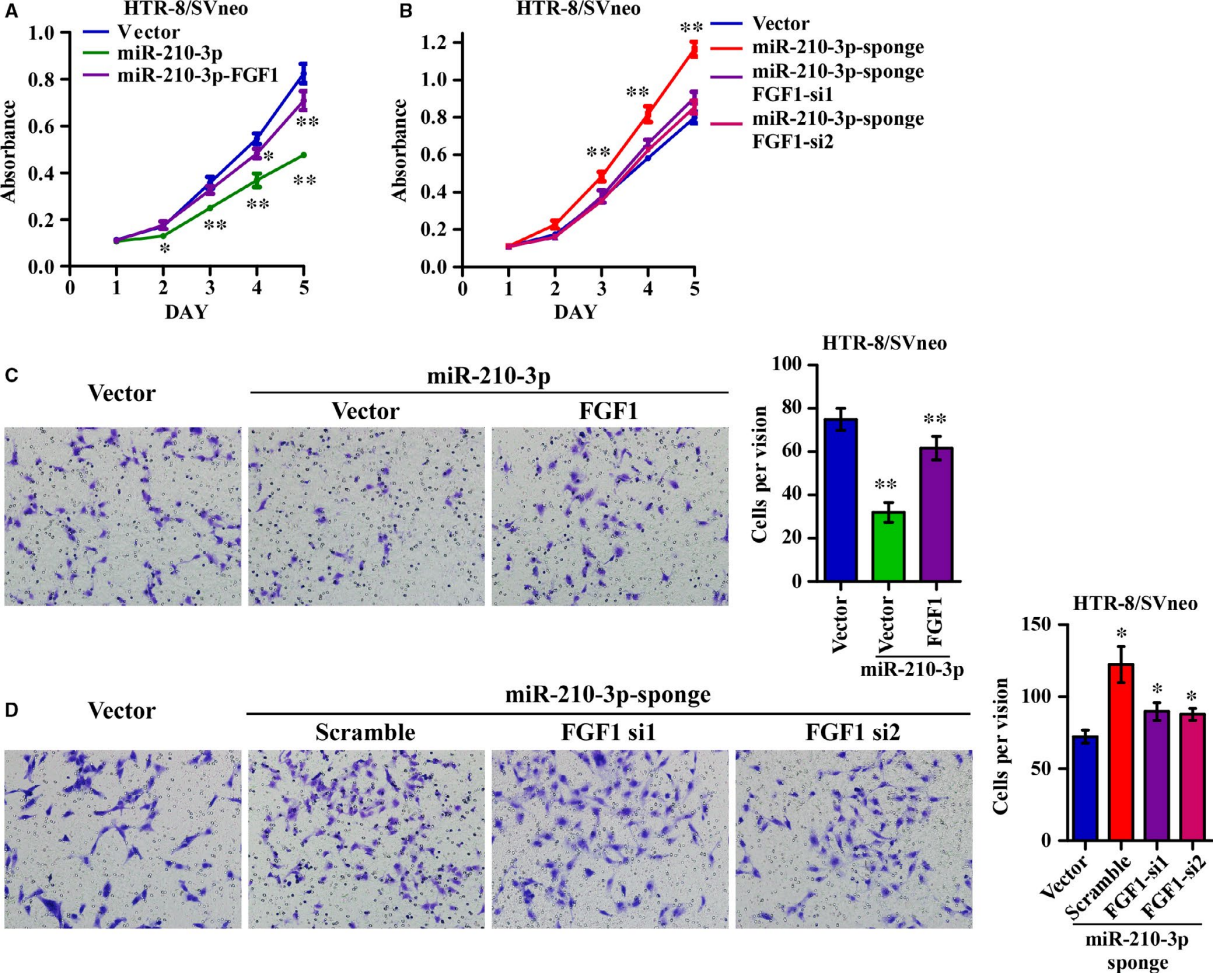
Fibroblast growth factor 1 (FGF1) is required for miR‐210‐3p‐mediated regulation of HTR‐8/SVneo cell proliferation and invasiveness. (A,B) Fibroblast growth factor 1 expression was restored in miR‐210‐3p‐overexpressing cells (A), and FGF1 expression was further silenced in cells transfected with the miR‐210‐3p sponge (B). Growth curves for the indicated cells were measured via MTT assay. (C,D) Fibroblast growth factor 1 expression was restored in miR‐210‐3p‐overexpressing cells (C), and FGF1 expression was further silenced in cells transfected with a miR‐210‐3p sponge (D). Representative images and quantification of invading cells in the Matrigel‐coated Transwell assay are shown as indicated. (*P < 0.05, **P < 0.01)

To further validate that miR‐210‐3p attenuated trophoblast proliferation and invasiveness by targeting FGF1, we restored FGF1 expression in miR‐210‐3p‐overexpressing HTR‐8/SVneo cells. As shown in Figure 4(A,C), restoring FGF1 expression partially rescued the proliferation and invasiveness of miR‐210‐3p‐overexpressing cells. However, silencing FGF1 with two effective siRNAs in cells transfected with the miR‐210‐3p sponge partially reversed the increases in proliferation and invasiveness, further supporting the possibility that FGF1 is a direct functional target of miR‐210‐3p (Figure 4B,D). Taken together, these results demonstrated that FGF1 downregulation was functionally important for the inhibitory effect of miR‐210‐3p on HTR‐8/SVneo cell proliferation and invasiveness.

### 3.5 Hypoxia regulates HTR‐8/SVneo cell growth and invasiveness through the HIF1α/miR‐210‐3p/FGF1 axis

**Figure 5:**
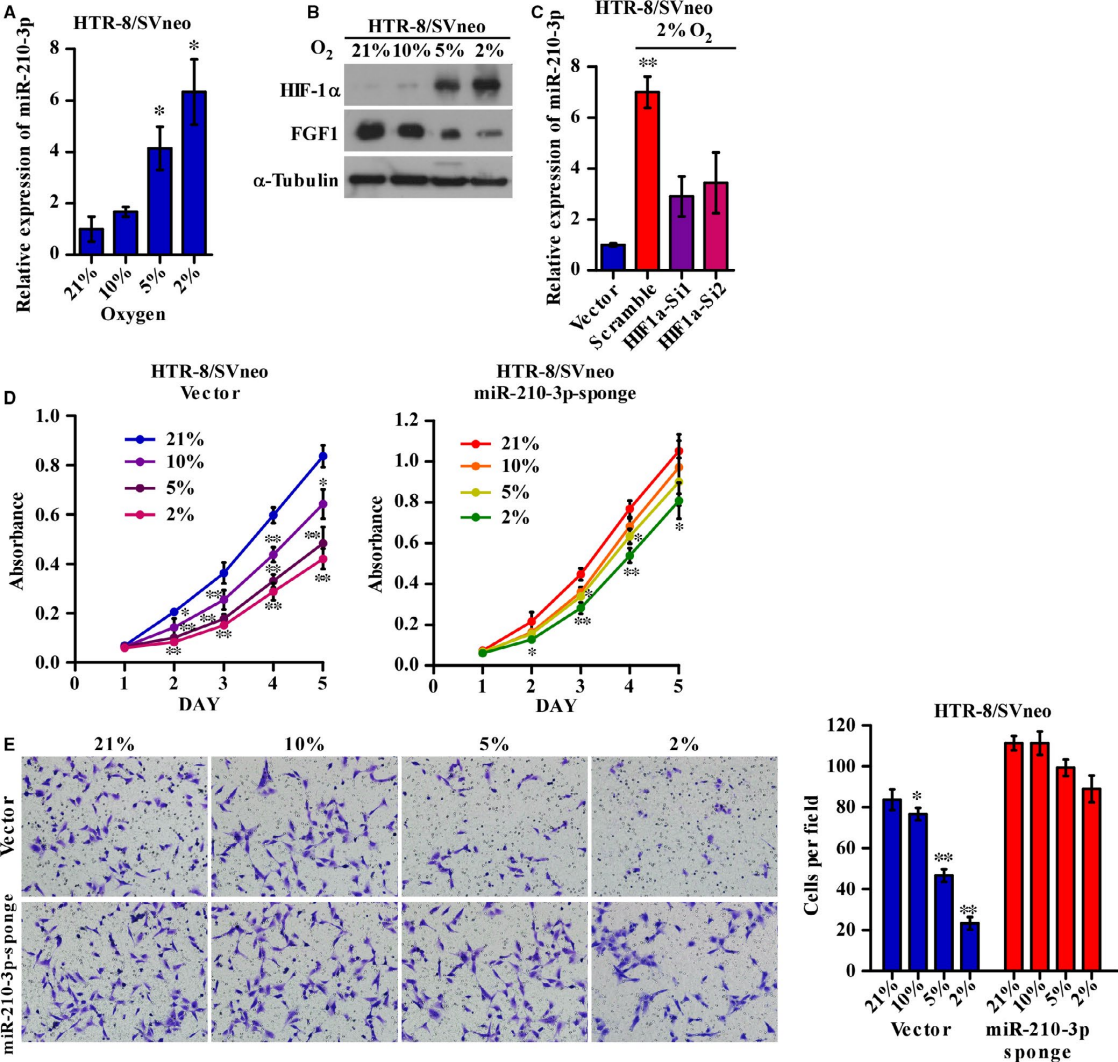
Hypoxia regulates HTR‐8/SVneo cell proliferation and invasiveness through the HIF1α/miR‐210‐3p/FGF1 axis. (A,B) HTR‐8/SVneo cells were incubated under normoxic conditions with 21% oxygen and under hypoxic conditions with 10%, 5% and 2% oxygen for 48 h. qRT‐PCR analysis of the relative expression of miR‐210‐3p (A), and Western blotting analysis of the protein levels of FGF1 and HIF1α (B) at the four different oxygen concentrations. (C) HIF1α was silenced by siRNAs in HTR‐8/SVneo cells. qRT‐PCR analysis of the relative expression of miR‐210‐3p in the indicated cells at 21% or 2% oxygen concentrations. (D,E) HTR‐8/SVneo cells containing the miR‐210‐3p sponge and corresponding vector were incubated under normoxic conditions with 21% oxygen and under hypoxic conditions with 10%, 5% and 2% oxygen for 48 h. Growth curves for the indicated cells at different oxygen concentrations were measured via MTT assay (D). Representative images and quantification of invading cells at different oxygen concentrations in the Matrigel‐coated Transwell assay are shown (E). (*P < 0.05, **P < 0.01). HIF1α, hypoxia‐inducible factor 1α; FGF1, fibroblast growth factor 1

MicroRNA‐210‐3p expression is strongly induced by HIF1α under hypoxic conditions. To explore the regulatory role of miR‐210‐3p during hypoxia, HTR‐8/SVneo cells were incubated under normoxic conditions with 21% oxygen and under hypoxic conditions with 10%, 5% and 2% oxygen for 48 hours. qRT‐PCR analysis revealed that miR‐210‐3p expression was upregulated at decreased oxygen concentrations (Figure 5A). Western blotting showed that the protein levels of HIF1α were significantly increased in the hypoxic group compared to those in the normoxic group, while the expression levels of FGF1 in the hypoxic group were downregulated at decreased oxygen concentrations (Figure 5B). In addition, the mRNA level of HIF1α did not change with the oxygen concentration or miR‐210‐3p expression level (Figure S1B,C). Moreover, silencing HIF1α with siRNAs strongly attenuated the induction of miR‐210‐3p expression during hypoxia, indicating that HIF1α is required for hypoxia‐induced miR‐210‐3p overexpression (Figure 5C). Furthermore, the proliferation and invasiveness of HTR‐8/SVneo cells under hypoxic conditions, especially in 2% oxygen, was significantly impaired, while inhibiting miR‐210‐3p largely rescued the loss of proliferation and invasion seen under hypoxic conditions (Figure 5D,E). These results indicated that hypoxic pre‐conditioning upregulated miR‐210‐3p expression levels via HIF1α and inhibited proliferation and invasion through the HIF1α/miR‐210‐3p/FGF1 axis in HTR‐8/SVneo cells.

### 3.6 Correlation of miR‐210‐3p expression with HIF1α and FGF1 expression in placental tissues

**Figure 6:**
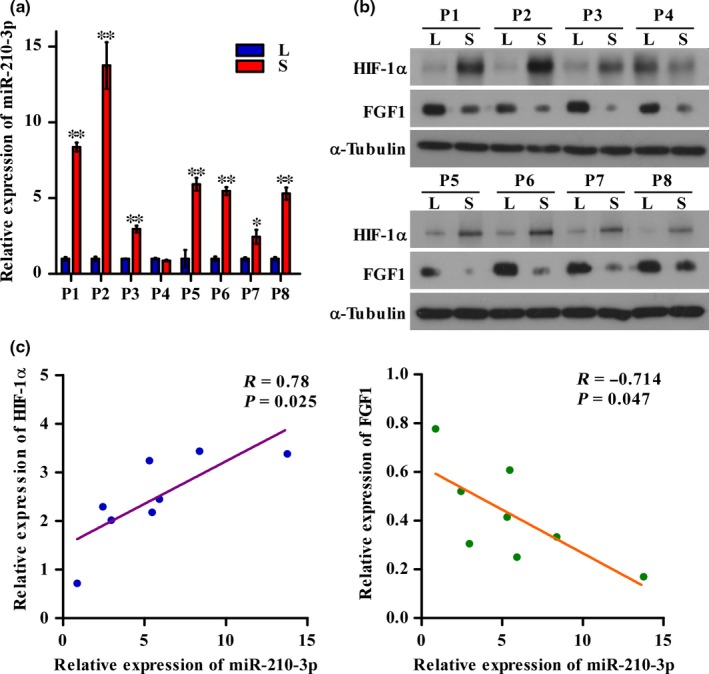
Correlation of miR‐210‐3p expression with hypoxia‐inducible factor 1α (HIF1α) and fibroblast growth factor 1 (FGF1) expression in placental tissues. A, qRT‐PCR analysis of the relative expression of miR‐210‐3p in placental tissues from eight pairs of selective intrauterine growth restriction (sIUGR) twins (P1‐P8). B, Western blotting analysis of the protein levels of FGF1 and HIF1α in placental tissues from eight pairs of sIUGR twins (P1‐P8). C, Correlation analysis of miR‐210‐3p expression with FGF1 and HIF1α expression. L, larger twin; S, smaller twin. (*P < 0.05, **P < 0.01)

As the in vitro experiments revealed that miR‐210‐3p expression was induced under hypoxia through HIF1α and inhibited FGF1 expression, we further examined the correlation between miR‐210‐3p expression and the protein levels of both FGF1 and HIF1α in the placental tissues of the smaller twins paired with those of the larger twins in sIUGR cases. As shown in Figure 6(A,B), significantly increased levels of HIF1α and miR‐210‐3p, and decreased levels of FGF1 were found in the smaller twins relative to those levels in the larger twins. The linear regression and correlation analyses showed that in the placental tissues, HIF1α expression was positively correlated with miR‐210‐3p expression, while FGF1 expression was negatively correlated with miR‐210‐3p expression, suggesting that the HIF1α/miR‐210‐3p/FGF1 axis is clinically relevant in sIUGR (Figure 6C).

## 4 DISCUSSION

As a pre‐requisite for the survival and development of the foetus, the placenta plays a critical role in the exchange of oxygen, nutrients and waste products between the mother and the foetus. Unequal division of the placenta is the most important cause of discordant growth in MC twins.^30^ Typically, the smaller twin has a lower placental share, which reflects limited interaction with the maternal circulation and restriction of the supply of oxygen and nutrients. We demonstrated that miR‐210‐3p expression was significantly upregulated in the placental share of the smaller foetuses and that the increased expression of miR‐210‐3p was strongly correlated with insufficient placentation by inhibiting the proliferation and invasive of trophoblast cells.

The processes of implantation and placentation largely depend on the invasiveness, differentiation and proliferation capabilities of trophoblasts. Several studies have shown that miR‐210 repressed trophoblast invasion and migration through its target genes. Zhang et al^31^ reported that miR‐210 attenuates trophoblast cell migration and invasion by repressing the expression of Homeobox‐A9 (HOXA9) and Ephrin‐A3 (EFNA3) in the human choriocarcinoma cell line JAR. Thrombospondin type I domain‐containing 7A (THSD7A) and potassium channel modulatory factor 1 (KCMF1) were also identified as targets of miR‐210 in HTR‐8/SVneo cells.^32^, ^33^ Lee et al^34^ found that iron‐sulphur cluster scaffold homolog (ISCU) was a target gene of miR‐210 in the Swan 71 and BeWo cell lines. The expression of all of these target genes was downregulated in pre‐eclamptic placentas compared to that in placentas from normal pregnancies. Moreover, increased expression of miR‐210 was detected in placental tissues from pregnancies with pre‐eclampsia.^31^, ^35^ Shallow placental implantation is thought to be the key fundamental pathological feature of both pre‐eclampsia and IUGR in singleton pregnancies.^36^, ^37^ Both placental volume and growth are reduced significantly between 12‐22 weeks in these two diseases.^38^

Our study identified that FGF1 is a functional target of miR‐210‐3p and is involved in the proliferation and invasiveness of trophoblasts. Although the molecular mechanism by which FGF1 regulates trophoblast function and placentation is largely unknown, several studies have reported that FGF1 is associated with pregnancy‐related diseases. Huang et al^39^ showed reduced expression of fibroblast growth factor receptor 1 (FGFR1) in placentas from foetuses affected by IUGR. Marwa et al^40^ reported that common genetic variants of the FGF1 allele, which may influence FGF1 activity, were associated with pre‐eclampsia pathogenesis. Hess et al^16^ found that FGF1 expression was significantly downregulated in decidualized endometrial stromal cells coincubated with conditioned medium from trophoblasts in vitro. However, further studies are needed to elucidate the functional roles of FGF1 in the proliferation and invasion capabilities of trophoblasts.

Defective trophoblast invasion leads to a relative hypoxic status in the partial placenta. Cells can adapt to low oxygen levels in part through the activation of HIF1α.^41^ Consistent with the results of previous reports, our results suggest that miR‐210‐3p levels in trophoblasts are strongly induced by HIF1α under hypoxia and play an important role in the decreased invasiveness capability of trophoblast cells, which in turn, contributes to hypoxia‐related pregnancy complications such as sIUGR and pre‐eclampsia. Interestingly, Kelly et al^42^ reported that miR‐210 expression results in HIF1α stabilization because of decreased levels of the enzyme glycerol‐3‐phosphate dehydrogenase 1‐like (GPD1L), which was identified as a novel regulator of HIF1α stability and a direct target of miR‐210. However, some findings suggest that hypoxia stimulated cell differentiation and angiogenesis, which are also critical for embryonic and placental development. Fasanaro et al^43^ reported that miR‐210 upregulation in human umbilical vein endothelial cells (HUVECs) results in increases in tubulogenesis and vascular endothelial growth factor‐induced cell migration through repression of its relevant target EFNA3. Furthermore, FGFl is recognized as a proangiogenic factor in vivo, and the release of FGFl has been shown to be induced by hypoxia.^44^ One explanation may be that hypoxia functions differently in different types of cells and biological processes. Nevertheless, the mechanisms of hypoxia underlying the onset and progression of sIUGR cannot be determined; in other words, whether hypoxia is the cause or the consequence of impaired trophoblast function remains unclear.

The correlations among miR‐210‐3p levels, HIF1α and FGF1 expression and the smaller placental share were validated in our sIUGR specimens, providing additional evidence of miR‐210‐3p involvement in the development of sIUGR. Moreover, clinical management and outcome are largely associated with the type of UA Doppler pattern in MC twins.^45^ Generally, type I sIUGR twins have a favourable outcome, with a survival rate of nearly 100%, while type II twins have the poorest prognosis. Our results indicated that the variance in miR‐210‐3p expression was larger in the placental share of the smaller foetus than in that of the larger foetus among type III twins. Type III cases with an intermittent absent end diastolic flow pattern have the most unequally shared placentas of the three types.^30^ Absent end diastolic UA flow is considered an indication of poor trophoblast invasion and reduced uteroplacental blood flow.^30^ Therefore, the placental expression of miR‐210‐3p might be partially connected to the characteristics on ultrasound examinations and prognosis of the pregnancy.

The limitation of our study is the lack of pairing of placental samples between the sIUGR group and normal MCDA group. Placental tissue was collected after delivery by elective caesarean section and the indications for delivery depend on maternal or foetal situations. Average gestational age at delivery was 32 weeks in sIUGR twins, while it was 35 weeks in normal MCDA twins. The expression level of miRNAs in placenta might differ among different gestational ages.

In summary, our data show that miR‐210‐3p is upregulated by hypoxia and disrupts the proliferation and invasion of trophoblasts and identify for the first time that FGF1 is an influential target of miR‐210‐3p. Moreover, we showed that FGF1 expression was negatively correlated with miR‐210‐3p expression in the placental share of the smaller twin in sIUGR pregnancies. These results demonstrate that the interaction between miR‐210‐3p and FGF1 is clinically relevant in these MC twins, who harbour an almost identical genetic background. Our findings not only indicate that miR‐210‐3p plays important regulatory roles in placentation and foetal development but also provide a novel epigenetic mechanism through which miR‐210‐3p may be associated with phenotypic discrepancies in monozygotic twins.

## CONFLICT OF INTEREST

The authors confirm that there are no conflicts of interest.

## AUTHOR CONTRIBUTIONS

Lin Li and Xuan Huang performed the experiments and drafted the manuscript. Zhiming He contributed to the collection of the samples. Yuanyan Xiong performed RNA‐seq and analysed the data. Qun Fang supervized the experiments and revised the manuscript. All authors approved the final manuscript.
