# Supplementary figures and images for: miRNA‐210‐3p regulates trophoblast proliferation and invasiveness through fibroblast growth factor 1 in selective intrauterine growth restriction
Source: J Cell Mol Med. 2019 Apr 16;23(6):4422–33. doi: 10.1111/jcmm.14335 (PMC6533475; doi:10.1111/jcmm.14335)

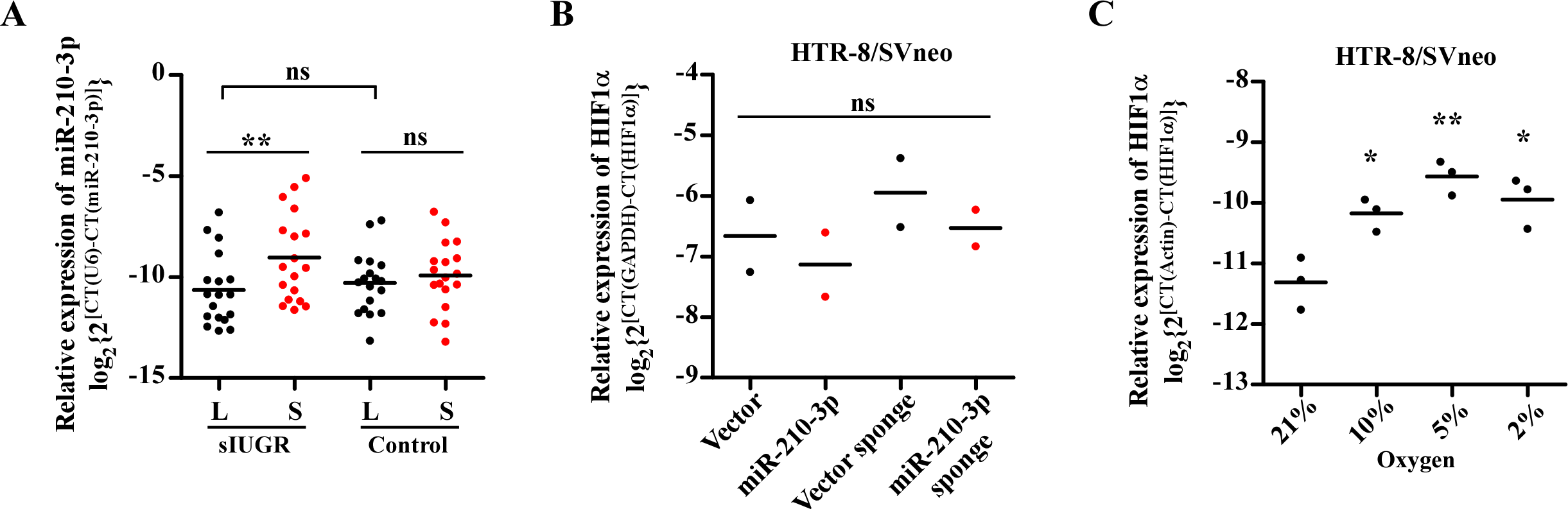

Supplement: Supplementary file 1 [file JCMM-23-4422-s001.tif]
