# Supplementary material for: miRNA‐210‐3p regulates trophoblast proliferation and invasiveness through fibroblast growth factor 1 in selective intrauterine growth restriction
Source: J Cell Mol Med. 2019 Apr 16;23(6):4422–33. doi: 10.1111/jcmm.14335 (PMC6533475; doi:10.1111/jcmm.14335)
Supplement: Supplementary file 2 [file JCMM-23-4422-s002.docx]

**Figure S1.** A, qRT-PCR results showing miR-210-3p levels in the placental share of the smaller fetus compared to those of the larger fetus in the sIUGR group and control group. The relative miR-210-3p expression was calculated using the following formula: log_2_{2^[CT(U6)-CT(miR-210-3p)]^}. B, qRT-PCR results showing HIF1α expression in HTR-8/SVneo cells stably overexpressing miRNA-210-3p, silenced miRNA-210-3p with sponge, or the corresponding vector controls. The relative HIF1α expression was calculated using the following formula: log_2_{2^[CT(GAPDH)-CT(HIF1α)]^}. C, qRT-PCR results showing HIF1α expression in parental HTR8/SVneo cells incubated in normal or hypoxia conditions. The relative HIF1α expression was calculated using the following formula: log_2_{2^[CT(Actin)-CT(HIF1α)]^}. L, larger twin; S, smaller twin. (* *P*<0.05, ** *P*<0.01)
